# Editorial: Green chemistry and the consumer: towards greener consumer products

**DOI:** 10.3389/fchem.2024.1546987

**Published:** 2025-01-07

**Authors:** James Clark

**Affiliations:** Green Chemistry Centre of Excellence, University of York, York, United Kingdom

**Keywords:** consumer, additives, biosuractant, nanometals, greener chemicals

Consumer concerns about chemicals generally and about particular adverse effects of chemicals in their products such as allergies, alongside increasing chemical legislation that is revealing ever-longer lists of undesirable chemicals, are putting manufacturers and retailers under pressure to remove some commonly used compounds from their products. These drivers for change coupled with an increasing desire for products derived from renewable rather than fossil resources makes the move towards green and sustainable chemistry in consumer goods irresistible. The chemistry community needs to create a “green chemistry toolbox” of chemicals that have a sustainable lifecycle and production process, are cost-effective and offer the range of properties desired by formulation scientists. These new products and formulations then have to be turned into actual products often with new supply chains being required. Real and cost-effective examples of green replacements are needed to help point the way towards safer consumer goods. The Research Topic Green Chemistry and the Consumer: Towards Greener Consumer Products was created to encourage original scientific articles and reviews that highlighted this need by describing new green chemical products suitable for incorporation into consumer good formulations, as well as expose the problems with existing products.

Cleaning is one of the very largest activities whereby ordinary people are exposed to chemicals and where the chemicals can be rapidly discharged into waste streams on use. The downstream ability of municipal sewage and other waste treatment plants, to effectively deal with these wastes is also a subject of great concern especially when so many of the chemicals used are synthetic and fossil based. In *Advances in the production of biosurfactants as green ingredients in home and personal care products*, the potential for biosurfactants is highlighted (Nasser et al.). Various microbial derived biosurfactants are critically evaluated for use in home and personal care products.

In *Sustainable biosurfactant production from secondary feedstock—recent advances, process optimization and perspectives*, the specific and highly sought after area of glycolipid biosurfactants is discussed in a review of their diverse applications including food processing, biomedical and increasing agricultural output (Miao et al.). The major issue of relatively high costs is addressed including the use of second-generation feedstocks.

The control of bacteria especially in terms of their impact on human health is a topic of massive concern worldwide. Traditionally medical antibiotics have been over-used and we are now experiencing increasing resistance to them making their efficacy less certain. There are also concerns about the very large volumes of antibiotics entering the environment. We need more weapons in our armoury and the particular example of nano-zinc oxide is described in the review article *Research on the antibacterial properties of nanoscale zinc oxide particles comprehensive review* and the associated Corrigendum (Nan et al.; Nan et al.). Metal nanoparticles are known to have great potential in microbial detection along with disease diagnosis and treatment. Zinc is an essential trace element crucial for human growth and development, and nano zinc oxide is a very appealing choice for anti-bacterials as is described and illustrated here.

The global concerns about plastic wastes has led to a steady shift away from plastics in some areas most notably for the consumer, in food packaging. The most commonly used alternative materials used by the food industry are paper and board but it is vital that we quickly study all potential impacts of these materials as soon as possible. We cannot afford to only discover problems when they have started to severely damage the environment and or human health. As with almost all materials, attention must be given to all their components meaning the presence of ubiquitous additives like plasticizers. In *Investigation of potential migratables from paper and board food contact materials*, the migrations of such substances from a variety of paper and board items including straws and takeaway food packaging are investigated (Mario et al.). The risks posed by some of these substances including bisphenols and amines are also discussed.

